# Retention in care and virological failure among adult HIV+ patients on second-line ART in Rwanda: a national representative study

**DOI:** 10.1186/s12879-019-3934-2

**Published:** 2019-04-05

**Authors:** Sabin Nsanzimana, Muhammed Semakula, Vedaste Ndahindwa, Eric Remera, Dieudonne Sebuhoro, Jean Paul Uwizihiwe, Nathan Ford, Marcel Tanner, Steve Kanters, Edward J. Mills, Heiner C. Bucher

**Affiliations:** 1 Institute of HIV Disease Prevention and Control, Rwanda Biomedical Centre, KG 203 St, Kigali, Rwanda; 2 Basel Institute for Clinical Epidemiology and Biostatistics, University Hospital Basel, University of Basel, Spitalstrasse 12, 1st floor, CH-4031 Basel, Switzerland; 3 Swiss Tropical and Public Health Institute, University of Basel, Socinstrasse 57, 4051 Basel, Switzerland; 4 University of Rwanda, School of Medicine and Allied Sciences, KK 737 Street-Gikondo, Kigali, Rwanda; 5 Centre for Infectious Disease Epidemiology and Research, School of Public Health and Family Medicine, Faculty of Health Sciences, University of Cape Town, Observatory, 7925 South Africa; 6 School of Population and Public Health, University of British Columbia, 2206 East Mall, Vancouver, BC V6T 1Z3 Canada; 7 Department of Health Research Methods, Evidence, and Impact, McMaster University, 1280 Main Street, West Hamilton, ON L8S 4K1 Canada

**Keywords:** HIV, Second-line antiretroviral therapy, Treatment failure

## Abstract

**Background:**

Currently, there is limited evidence on the effectiveness of second-line antiretroviral therapy (ART) in sub-Saharan Africa. To address this challenge, outcomes of second-line protease inhibitor (PI) based ART in Rwanda were assessed.

**Methods:**

A two-stage cluster sampling design was undertaken. 49 of 340 health facilities linked to the open-source electronic medical record (EMR) system of Rwanda were randomly sampled. Data sampling criteria included adult HIV positive patients with documented change from first to second-line ART regimen. Retention in care and treatment failure (viral load above 1000 copies/mL) were evaluated using multivariable Cox proportional hazards and logistic regression models.

**Results:**

A total of 1688 patients (60% females) initiated second-line ART PI-based regimen by 31st December 2016 with a median follow-up time of 26 months (IQR 24–36). Overall, 92.5% of patients were retained in care; 83% achieved VL ≤ 1000 copies/ml, 2.8% were lost to care and 2.2% died. Defaulting from care was associated with more recent initiation of ART- PI based regimen, CD4 cell count ≤500 cells/mm^3^ at initiation of second line ART and viral load > 1000 copies/ml at last measurement. Viral failure was associated with younger age, WHO stage III&IV at ART initiation, CD4 cell count ≤500 cells/mm^3^ at switch, atazanavir based second-line ART and receiving care at a health center compared to hospital settings.

**Conclusions:**

A high proportion of patients on second-line ART are doing relatively well in Rwanda and retained in care with low viral failure rates. However, enhanced understandings of adherence and adherence interventions for less healthy individuals are required. Routine viral load measurement and tracing of loss to follow-up is fundamental in resource limited settings, especially among less healthy patients.

## Background

According to the 2016 estimates of the Joint United Nations Programme on HIV/AIDS (UNAIDS), 36.7 million people lived with HIV globally, and approximately half were on antiretroviral therapy (ART) [1, 2]. Sub-Saharan Africa (SSA), in particular, accounts for more than 80% of the global population of people living with HIV (PLHIV) [3]. Since the introduction of ART in 1996, there have been substantial declines in morbidity and mortality related to HIV [3, 4]. Despite this achievement, a considerable number of people have failed to maintain a sustained virological and immunological response to ART [5].

The World Health Organization (WHO) recommends the use of a two nucleoside/nucleotide reverse transcriptase inhibitors (NRTIs) backbone and a non-nucleoside/nucleotide reverse transcriptase inhibitor (NNRTI) as a first-line ART; The proposed switch to a second-line regimen comprises of a ritonavir-boosted protease inhibitor (PI/r) with two NRTIs [6]. The number of PLHIV switching from first- to second-line ART regimens is increasing and this shift is related to several factors. Primarily, non-adherence to medication, as a result of, adverse events or non-continuous medication access was reported in many studies as the key cause of treatment failure [7]. Additionally, as ART scale-up in SSA and elsewhere was initiated in 2004 [3, 4], more PLHIV began receiving treatment. Unavoidably, even under optimal circumstances, treatment failures will occur overtime, resulting in an increase in the number of individuals in requiring second-line ART. Other factors, such as increased resistance testing, improved adverse events detection and enhanced country access to affordable medications will also likely contribute to improved accessibility to second-line ART [4].

Since the launch of second-line regimens in SSA, outcomes of large-scale national ART programmes were assessed only in a few studies [8–13]. Botswana and Rwanda are two countries in SSA achieving the highest ART coverage > 80% [1, 2]. Of the estimated 220,000 PLHIV in Rwanda, 175,398 (80%) were receiving ART by December 2016 [9]. Rwanda, for example, has demonstrated a high rate of patients on first-line ART (> 90%) [14]. Similarly, the number of patients on second-line ART in Rwanda has also increased substantially in the last decade from 388 patients in 2007 to 7625 by the end of December 2016, representing ~ 4% of all patients on ART. Given this progression, the purpose of this study is to assess the outcomes associated with the rapid expansion of second-line ART access in Rwanda [15].

## Methods

### Study design and data sources

By the end of 2016, a total of 553 health facilities were offering HIV treatment in Rwanda. Among them, 513 had enrolled 7625 patients on second-line ART. Since the open-source electronic medical record (EMR) system was available in only 340 of the 553 health facilities, this constituted our sampling frame. A two-stage cluster sampling design was undertaken to randomly select 49 of the 340 eligible sites where all patients were considered for analysis in a retrospective observational cohort study using routinely collected program data.

A comprehensive list of health facilities providing second-line ART in Rwanda was compiled using the Health Management Information data hosted at the Rwanda Biomedical Center. This list formed the basis of our first sampling frame, which consisted of our randomly selected sample of health facilities. We restricted our selection to health facilities that had a fully functioning EMR system. There were no other expected differences with health facilities that were in the process of EMR roll out. Our stratification was balanced, enabling equal opportunity for the inclusion of urban, rural, small and big sites.

Our data source consists of electronic medical records. After selecting 49 sites, we determined the data sampling criteria to include all adult patients (aged 15 years or older) on second-line ART. The total number of patient data used for the study was 1689, representing about 25% of the total patients on second-line regimen in Rwanda. We excluded from our analysis all patients who had switched to third line ART. Since Rwanda is currently in the process of transferring HIV related data sources to a national electronic database, some data are still stored locally. Two authors DS and VN visited all 49 health facilities and extracted data backups from EMR local servers using *mysql* software and exported them to STATA version 14 to conduct the analyses.

### Study population and definitions

Our study included patients aged 15 years or older, whoever switched to second-line ART in 49 randomly selected health facilities in Rwanda since the start of second line program in 2004 until 31st December 2016.

First-line ART regimens were composed of one NNRTI plus two NRTIs and second-line regimens were PI-based, in accordance with national guidelines. Two key possible reasons for change may be due to first-line treatment failure (virological and/or immunological) or the result of adverse-effects to any compound in first-line combinations or prior exposure to antiretroviral drugs. The frequency of CD4 cell count measurement was bi-annual while viral load has been measured annually for most of patients according to the national guidelines except in exceptional circumstances guided by decision from individual clinician.

We defined *virological failure* as having a viral load (VL) >  1000 copies/mL after at least 12 months on second-line ART with self-reported good adherence to medication (> 90% no dose missed). Viral load failure was used as an approach to confirm *treatment failure*. The VL suppression threshold of ≤1000 copies/mL and *undetectable VL* < 20 copies/mL were in accordance with the 2016 WHO consolidated guidelines on the use of antiretroviral drugs for treating and preventing HIV infection [6].

We defined *retention* as alive and in care on second-line ART at the time of data collection (31st December 2016) and with no met criteria for loss to follow-up (having missed contact with the health facility during 3 consecutive months). Deaths were assessed using recorded medical data in the EMR, which included deaths that occurred outside of the health facilities. Deaths were recorded within the national mortality registry. Both viral suppression and loss to care served as the outcomes. The explanatory variables for this analysis were all measured at time of switch from first to second line ART and included demographic variables (age, sex, marital status, body mass index (BMI), clinical variables (TB screening status, CD4 cell count, WHO stage, viral load, date of ART initiation, type of ART regimen), and health facility-level variables (type of health facility: district hospitals, health centers and referral hospitals).

### Statistical analyses

Data are presented as medians and interquartile ranges (IQRs) for continuous variables and frequencies and percentages for categorical variables. Fisher’s exact test was used to assess the association between outcome of interest (retention and viral load suppression) and each predictor. We used multivariate Cox proportional-hazards regression to analyze time to discontinuation (loss to follow-up or death) on second-line ART. The regression model included the following covariates: age, gender, CD4 cell count strata, WHO clinical stage, ART regimens, viral load, and type of health facilities at the time of first ART. The overall dataset contained only one case of missing value which was not considered for retention outcome. We controlled all different antiretroviral backbones and PI based combinations for each individual patient to assess differences in ART formulations vis-à-vis retention and viral suppression.

The proportional hazard model test was used to ensure that the proportional assumption was met. For model selection, we used Aikaike Information Criteria (AIC) to identify the model that best-balanced parsimony and minimized residuals.

To model virological suppression, multiple logistic regression was used to analyze viral load suppression using the latest viral measurements. Finally, we calculated the probability of a subject not being suppressed given a set of predictors in order to obtain adjusted coefficients. The coefficients were expressed as adjusted odds ratios (OR). The model diagnostics were performed to assess the goodness of fit using Hosmer and Lemeshow test, deviance, and Pearson’s Statistics. All analyses were conducted using STATA statistical software, version 14.

### Ethical approval

Data used for this study were anonymized, de-identified and routinely collected programme data maintained by the Rwanda Biomedical Centre, Division of HIV/AIDS, STIs and Other Blood Borne Infections. No participants were involved directly in the data collection therefore their consent was waived by the Rwanda National Ethics Committee which also approved the use of routine programme data presented here. The Rwanda Ministry of Health also granted approval for data access and use to the principal investigator (SN) for the purposes of improving programme performance in Rwanda.

## Results

**Fig. 1:**
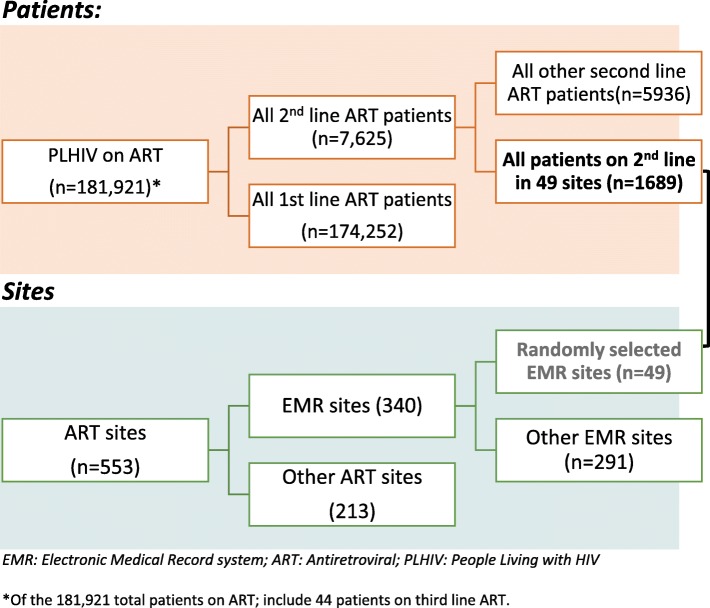
Flow diagram of Second line ART in Rwanda study sites and patients selection process

Among the 181,921 individuals on ART in Rwanda [9], 174,252 (95.8%) received first-line ART and 7625 (4.2%) received second-line ART by 31st December 2016 while 44 patients were on third line ART [16]. Figure 1 presents a flow diagram of the second line ART Rwanda study sites and the patient selection process.

In our analysis, 1688 eligible patients were included, all of which had initiated second-line ART in 49 randomly selected health facilities representing about 25% of all patients on second-line ART nationwide. Table 1 presents the baseline characteristics of the selected patients. There were more women (60%) in the sample.

**Table 1:** Baseline characteristics of patients on second-line ART in Rwanda stratified by retention and viral suppression

Characteristics	Total	Retained	Defaulting from care		Viral load suppression (copies/ml)		
		Alive	LTFU	Died	Total	≤ 1000	> 1000
	n (%)	n (%)	N (%)	n (%)	n (%)	n (%)	n (%)
Median age (IQR)	41 (33,49)						
Age category(year)							
15–29	327 (19)	300 (92)	24 (7)	3 (1)	327 (19)	242 (74)	85 (26)
30–39	426 (25)	390 (92)	28 (7)	8 (2)	426 (25)	344 (81)	82 (19)
40–59	841 (50)	785 (93)	35 (4)	21 (2)	842 (50)	718 (85)	124 (15)
60+	94 (6)	87 (93)	2 (2)	5 (5)	94 (6)	84 (89)	10 (11)
Total	1688 (100)	1562 (93)	89 (5)	37 (2)	1689 (100)	1388 (82)	301 (18)
Sex							
Female	1031 (61)	952 (92)	55 (5)	24 (2)	1032 (61)	866 (84)	166 (16)
Male	657 (39)	610 (93)	34 (5)	13 (2)	657 (39)	522 (79)	135 (21)
Total	1688 (100)	1562 (93)	89 (5)	37 (2)	1689 (100)	1388 (82)	301 (18)
Marital status							
Single	344 (20)	309 (90)	27 (8)	8 (2)	344 (20)	259 (75)	85 (25)
Married/Cohabitating	648 (38)	602 (93)	30 (5)	16 (2)	649 (38)	540 (83)	109 (17)
separated/Divorced	98 (6)	89 (91)	7 (7)	2 (2)	98 (6)	86 (88)	12 (12)
Widowed	226 (13)	214 (95)	6 (3)	6 (3)	226 (13)	198 (88)	28 (12)
Missing	372 (22)	348 (94)	19 (5)	5 (1)	372 (22)	305 (82)	67 (18)
Total	1688 (100)	1562 (93)	89 (5)	37 (2)	1689 (100)	1388 (82)	301 (18)
ART Initiation period							
2009 and before	1062 (63)	1004 (95)	34 (3)	24 (2)	1063 (63)	892 (84)	171 (16)
2010–2012	502 (30)	447 (89)	44 (9)	11 (2)	502 (30)	400 (80)	102 (20)
2013–2016	123 (7)	110 (89)	11 (9)	2 (2)	123 (7)	96 (78)	27 (22)
Total	1687 (100)	1561 (93)	89 (5)	37 (2)	1688 (100)	1388 (82)	300 (18)
TB Screening							
Negative	1458 (86)	1356 (93)	75 (5)	27 (2)	1459 (86)	1213 (83)	246 (17)
Positive	151 (9)	138 (91)	8 (5)	5 (3)	151 (9)	111 (74)	40 (26)
N/A	79 (5)	68 (86)	6 (8)	5 (6)	79 (5)	64 (81)	15 (19)
Total	1688 (100)	1562 (93)	89 (5)	37 (2)	1689 (100)	1388 (82)	301 (18)
Body mass index							
Normal weight	964 (57)	894 (93)	50 (5)	20 (2)	965 (57)	783 (81)	182 (19)
Underweight	186 (11)	165 (89)	13 (7)	8 (4)	186 (11)	144 (77)	42 (23)
overweight & Obese	538 (32)	503 (93)	26 (5)	9 (2)	538 (32)	461 (86)	77 (14)
Total	1688 (100)	1562 (93)	89 (5)	37 (2)	1689 (100)	1388 (82)	301 (18)
Median CD4 (IQR)	418 (248, 618)						
> 500 cells/mm^3^	638 (38)	606 (95)	26 (4)	6 (1)	638 (38)	599 (94)	39 (6)
≤ 500 cells/mm^3^	1050 (62)	956 (91)	63 (6)	31 (3)	1051 (62)	789 (75)	262 (25)
Total	1688 (100)	1562 (93)	89 (5)	37 (2)	1689 (100)	1388 (82)	301 (18)
WHO stage							
Stage 1–2	1042 (62)	971 (93)	51 (5)	20 (2)	1042 (62)	881 (85)	161 (15)
Stage 3–4	623 (37)	570 (91)	36 (6)	17 (3)	624 (37)	489 (78)	135 (22)
missing	23 (1)	21 (91)	2 (9)	0 (0)	23 (1)	18 (78)	5 (22)
Total	1688 (100)	1562 (93)	89 (5)	37 (2)	1689 (100)	1388 (82)	301 (18)
Second-line ARV regimen							
LPV/r + dual NRTI	1097 (65)	1011 (92)	55 (5)	31 (3)	1098 (65)	920 (84)	178 (16)
ATV/r + dual NRTI	591 (35)	551 (93)	34 (6)	6 (1)	591 (35)	468 (79)	123 (21)
Total	1688 (100)	1562 (93)	89 (5)	37 (2)	1689 (100)	1388 (82)	301 (18)
Median VL (IQR)	20 (20, 100)						
Viral load results							
≤ 20 copies/mL	1056 (63)	1002 (95)	43 (4)	11 (1)			
21–1000 copies/mL	331 (20)	303 (92)	20 (6)	8 (2)			
> 1000 copies/mL	301 (18)	257 (85)	26 (9)	18 (6)			
Total	1688 (100)	1562 (93)	89 (5)	37 (2)			
Retention							
Retained					1562 (93)	1305 (84)	257 (16)
Not retained					126 (7)	82 (65)	44 (35)
Total					1688 (100)	1387 (82)	301 (18)

*VL* viral load, *TB* tuberculosis, *BMI* body mass index, *ART* antiretroviral therapy, *WHO* World Health Organization, *ATV/r* ritonavir boosted atazanavir, *LPV/r* ritonavir boosted lopinavir, *NRTI* nucleoside/nucleotide reverse transcriptase inhibitors, *IQR* interquartile range, *LTFU* lost to follow-up

The median age range was 35–44 years and approximately 38% of patients had initiated ART at WHO stage 3 or 4. The majority of patients (64%) had initiated second-line ART prior to 2010 resulting in median follow-up time of 26 months (IQR 24–36).

In total, 1562/1688 (92.5%) individuals were retained in care, 126 (7.5%) were loss to follow-up (5.2%) or had died (2.2%). Retention appeared to be lowest among those aged 25–34 years, who were single, and the least healthy. Retention was 89.0% in individuals who were underweight and 90.7% among patients screened positive for tuberculosis, and 90.4% among those with CD4 cell counts below 350 cells/mm^3^ at initiation of ART.

In all 1688 individuals, at least one viral load result was available, regardless of whether patients had subsequently defaulted from care. Of these, 1387 of 1688 (83%) individuals were virologically suppressed (≤ 1000 copies/ml) at last follow-up, whereas 1056 (63%) achieved undetectable viral loads (≤ 20 copies/ml). Virological failure with VL >  1000 copies/mL was found in 301 of 1689 (18%) individuals. Of all those retained in care, a higher proportion were virologically undetectable at the time of the last available viral load test result compared to those lost to care (64% vs 43%). Virological failure was also higher among those who were lost to care (35% vs 16%). Retention in care was 94% for those who were virologically suppressed and 85% for those who were not suppressed.

### Hazards for defaulting from care

The predictors of defaulting from care are presented in Table 2.

**Table 2:** Predictors of attrition and virological failure on second-line ART

Predictors	Multivariate analysis			
	Defaulting from care		Virological failure	
	Adjusted hazard ratio (95% CI)	*p*-value	Adjusted odds ratio (95% CI)	*p*-value
Age category (years). Reference category Age 40–59 ^a^				
15–29 years	0.85 (0.42, 1.71)	0.654	2.22 (1.46, 3.38)	< 0.001
30–39 years	1.03 (0.58, 1.81)	0.930	1.45 (1.03, 2.05)	0.032
60+ years	1.87 (0.80, 4.38)	0.147	0.74 (0.36, 1.53)	0.421
Sex ^a^				
Male vs. female	0.87 (0.53, 1.43)	0.585	1.07 (0.80, 1.43)	0.644
Marital status. Reference category single ^a^				
Married/Cohabitating	0.48 (0.26, 0.90)	0.023	0.96 (0.64, 1.45)	0.857
Separated/Divorced	0.69 (0.24, 1.96)	0.487	0.56 (0.27, 1.14)	0.110
Widowed	0.57 (0.24, 1.34)	0.197	0.71 (0.40, 1.26)	0.242
Missing	0.31 (0.15, 0.65)	0.002	1.02 (0.68, 1.54)	0.916
ART initiation. Reference category 2009 and before ^a^				
2010–2012	2.43 (1.47, 4.01)	0.001		
2013–2016	2.49 (1.00, 6.18)	0.049		
TB Screening. Reference category TB negative +				
Positive	1.26 (0.64, 2.49)	0.504	1.40 (0.92, 2.13)	0.117
No screening	3.04 (1.49, 6.22)	0.002	1.01 (0.54, 1.88)	0.979
BMI category. Reference category recommended weight +				
Underweight BMI<	1.94 (1.05, 3.57)	0.034	0.99 (0.66, 1.50)	0.965
Overweight BMI >	1.23 (0.73, 2.06)	0.432	0.80 (0.59, 1.09)	0.155
CD4 Category +				
≤ 500 copies vs. > 500 cells/mm^3^	2.12 (1.20, 3.75)	0.009	5.40 (3.75, 7.77)	< 0.001
WHO Stages. Reference category Stage 1–2 ^a^				
Stage 3–4	1.22 (0.77, 1.96)	0.399	1.56 (1.18, 2.06)	0.002
Missing	0.49 (0.06, 3.73)	0.489	1.36 (0.46, 3.99)	0.574
Second-line regimen +				
ATV/r + 2 NRTI vs. LPV/r + 2 NRTI	0.52 (0.29, 0.93)	0.027	1.48 (1.12, 1.95)	0.005
Viral load suppression +				
> 1000 copies/mL vs ≤ 1000 copies/mL	2.95 (1.83, 4.76)	< 0.001		
Health facility types +				
HC vs. RH/PV	0.93 (0.52, 1.65)	0.799	1.55 (1.11, 2.17)	0.010
DH vs. RH/PV	1.23 (0.68, 2.22)	0.496	0.92 (0.63, 1.34)	0.653

^a^ variable measured at initiation of ART, **+** variable measured at switch to second line ART

*CI* confidence interval, *DH* district hospital, *HC* health centre, *PH* provincial hospital, *RH* regional hospital, *TB* tuberculosis, *BMI* body mass index, *ART* antiretroviral therapy, *WHO* World Health Organization, *ATV/r* ritonavir boosted atazanavir, *LPV/r* ritonavir boosted lopinavir, *NRTI* nucleoside/nucleotide reverse transcriptase inhibitors

BMI categories: underweight ≤18.5 kg/m^2^, normal weight = 18.5–24.9 kg/m^2^, overweight& obesity =25 kg/m^2^ or greater

These included ART initiation period from 2010 to 2012 and from 2013 to 2016 relative to 2009 or earlier (adjusted hazard ratios [HR] 2.43, 95% confidence interval [CI] 1.47–4.01 and HR 2.49, 95% CI 1.00–6.18), CD4 cell count ≤500 cells/mm^3^ vs CD4 cell count > 500 cells/mm^3^ at initiation (HR 2.12, 95% CI 1.20–3.75), lopinavir/ritonavir (LPV/r) vs. atazanavir/ritonavir (ATV/r) based second-line regimen (HR1.91, 95% CI 1.08–3.40) and viral load > 1000 copies/ml vs ≤ 1000 copies/ml at latest measurement (HR 2.60, 95% CI 1.71–3.94). In addition to these clinical variables, being married or cohabitating with a partner, relative to being single, was protective of defaulting from care (HR 0.48, 95% CI 0.26–0.90).

### Risk factors for virological failure

The following risk factors were associated with virological failure in multivariate analysis: Age groups 15–29 years and 30–39 years compared to age group 40–59 years (adjusted odds ratios (OR): 2.22, 95% CI 1.46–3.38 and OR 1.45, 95% CI 1.03–2.05), CD4 cell count ≤500 cells/mm^3^ vs. CD4 count > 500 cells/ mm^3^ at ART initiation (OR 5.40, 95% CI: 3.75–7.77), WHO stage III & IV care compared to WHO stage I and II at programme enrollment (OR, 1.56, 95% CI 1.18–2.06), ATV/r compared to LPV/r based second-line regimen (OR 1.48, 95% CI 1.12–1.95) and receiving care at a health center relative to regional or provincial hospital (OR 1.55, 95% CI 1.11–2.17).

## Discussion

Our study is the first to report on retention and viral load outcomes using a national representative sample of second-line ART patients in Rwanda. We found that, overall, a high proportion of patients were retained in care after a median follow-up of 26 months. The estimated 92.5% retention was higher than that reported in previous studies in similar settings [10, 17]. High retention in care in the Rwanda HIV programme was also previously reported [9, 18]. Several possible reasons for high rates of retention in care include the highly decentralized health system that provides easy to access HIV services (98% of health facilities in Rwanda offer integrated, comprehensive HIV services). Further, there is a strong network of PLHIV that supports peer adherence to medication including home visits, awareness and education activities in the communities. In addition, Rwanda has a robust electronic monitoring and surveillance system that allows early warning signs of lost to care, which initiates home visits by health care providers. Finally, health care seeking behavior in the Rwandan PLHIV population is high [9].

Our study identified key factors associated with defaulting from care: initiating at higher viral loads, low CD4 cell count, less clinical engagement, and time of treatment switch. Other studies from SSA and Asia have investigated predictors of attrition in care [10, 19–22]. Across many settings, loss to follow-up on second-line patients was significantly higher among those with low CD4 cell count at baseline, and previously undiagnosed treatment failure on a first-line regimen [20–29]. Other findings in similar settings reported that advanced disease at initiation was associated with attrition on second-line ART, and is likely the result of mortality [30], as well as higher viral load and age [20]. Studies from Malawi [21], Thailand [22] and South Africa [24] reported that adherence was the major determinant of treatment failure.

In this study, viral suppression rate (VL ≤ 1000 copies/mL) among second-line patients in Rwanda was estimated to be 83%. This rate is consistent with similar results observed in other resource limited settings with an average of 80% viral suppression after 12 months on second-line ART [19, 31]. Results obtained from this study are generally consistent with other studies in developing countries. For example, the pooled proportion of virological failure in a recent systematic review and meta-analysis on second-line ART in low- and middle-income countries was 23.1, 26.7 and 38.0% at 12, 24 and 36 months, respectively [18]. In many settings, virological failure was observed in the first 6 months following second-line ART start [18]. However, the reported results had large variations between studies and comparison of treatment failure might be difficult due to different cut offs used for viral load suppression across countries [32, 33].

Patients who were lost to care were more likely to be viraemic than patients who were retained in care – a finding observed also by others [34]. This has important implications for evaluating progress towards the UNAIDS 90–90-90 targets. For instance, assessing virological suppression (the third 90) only among patient retained in care will overestimate success unless losses to care are taken into account.

In Rwanda, a previous study reported that only 23% of patients presenting virological failure (> 1000 copies/mL) had drug resistance mutations suggestive of third line ART, though 77% with high viral load could still remain on efficacious second-line therapy [18, 35]. This reinforces the need for intensive adherence for patients presenting suboptimal viral load suppression before switching to costly and complicated salvage therapies. The same challenge was also reported in other resource-limited countries [19, 29, 35].

The HIV programme in Rwanda has made major shifts since 2009, when no new drug classes were available for cases of virological failure [20]. The new recommendation was implemented in 2013, when LPV/r based regimens were replaced by ATV/r based regimens. In our analysis, patients who started anti-retrovirals after 2010 had better retention on ART, yet no better VL outcomes.

For this study, we controlled the distributions of backbones for both LPV/r and ATV/r. There was equal distribution of zidovudine, tenofovir and abacavir for each PI-based combination; lamivudine was maintained across all second-line regimens as per national guidelines. Patients treated with ATV/r were significantly more likely to experience virological failure. A recent systematic review of six randomized controlled trials on the comparative efficacy of second-line ART did not find a difference in efficacy between LPV/r or ATV/r plus two NRTIs and LPV/r with raltegravir. Although ATV/r had a greater numerical efficacy compared to LPV/r, differences were not statistically significant [25]. Another study conducted in Uganda [26] compared LPV/r and ATV/r in patients failing first line ART (2NRTI + 1NNRTI) and confirmed comparable potency and efficacy. Being on LPV/r was twofold associated with defaulting from care, which could be due to the higher pill burden and adverse gastro-intestinal drug reactions associated to LPV/r causing low adherence to medication [36]. A recent study in Malawi among patients receiving ATV/r also reported that bilirubin levels predicted VL failure [27]. ATV/r prescription with related increased bilirubin has been associated with high interpatient disparities with hyperbilirubinemia and jaundice resulting into premature discontinuation of atazanavir and subsequently impact on virological outcome [28].

A major strength of our analyses is the relatively large sample size corresponding to 25% of all people living with HIV on second-line ART in Rwanda. We also used routinely reported data that reflects more precisely the everyday life of patients. In addition, we had few missing data and all our patients had at least one viral load measured in the last 12 months on ART. Data were collected from a diverse population of patients in large, medium and small sites. In addition, we managed to successfully demonstrate how nationwide routine surveillance open MRS data could be used to inform on patients’ retention and viral load suppression.

Our study also has several limitations. First, the data was collected from an open electronic medical record system for which individual patient-level data were routinely reported. As such, not all desired variables were available – most importantly adherence to HIV medication and outcomes among those lost to care. Second, we could not distinguish reasons for switching to second-line ART other than virological failure. Third, the sampling population only included 340 of the 513 health facilities with second-line patients. Thus, there is a risk of selection bias innate to the data availability. Finally, as with all observational studies, confounding through unmeasured covariates need to be considered when interpreting the reported associations.

## Conclusions

In conclusion, our study suggests that patients on second-line ART within Rwanda are doing relatively well, with high levels of retention in care and viral suppression. A better understanding of adherence and adherence interventions for those that are less healthy is required. Importantly, routine viral load measurement and tracing of loss to follow-up is fundamental in resource limited settings in order to minimize the risk of treatment failure.

## Abbreviations

AIC: Aikaike Information Criteria
ART: Anti-retroviral therapy
ATV/r: Atazanavir/ritonavir
BMI: Body mass index
CI: Confidence interval
EMR: Electronic medical record
HR: Hazard Ratio
IQRs: Interquartile ranges
LTV/r: Lopinavir/ritonavir
NNRTI: Non-nucleoside/nucleotide reverse transcriptase inhibitors
NRTIs: Nucleoside/nucleotide reverse transcriptase inhibitors
OR: Odds ratio
PI: Protease Inhibitor
PI/r: ritonavir-boosted protease inhibitor
PLHIV: People living with HIV
SSA: Sub-Saharan Africa
UNAIDS: Joint United Nations Programme on HIV/AIDS
VL: Viral load
WHO: World Health Organization
